# Interfacial Defects Dictated In Situ Fabrication of Yolk–Shell Upconversion Nanoparticles by Electron‐Beam Irradiation

**DOI:** 10.1002/advs.201800766

**Published:** 2018-07-25

**Authors:** Jin Xu, Datao Tu, Wei Zheng, Xiaoying Shang, Ping Huang, Yao Cheng, Yuansheng Wang, Xueyuan Chen

**Affiliations:** ^1^ CAS Key Laboratory of Design and Assembly of Functional Nanostructures State Key Laboratory of Structural Chemistry, and Fujian Key Laboratory of Nanomaterials Fujian Institute of Research on the Structure of Matter Chinese Academy of Sciences Fuzhou Fujian 350002 China

**Keywords:** electron‐beam irradiation, in situ fabrication, interfacial defects, yolk–shell upconversion nanoparticles

## Abstract

Homogeneous core–shell structured nanoparticles (NPs) are prevailingly designed to accommodate lanthanide emitters, and such an epitaxial shell deposited on core NP is generally believed to help eliminate surface traps or defects on the as‐prepared core. However, upon electron‐beam irradiation to core–shell–shell NaLuF_4_:Gd/Yb/Er@NaLuF_4_:Nd/Yb@NaLuF_4_ upconversion NPs (UCNPs), it is revealed that interfacial defects actually exist at the core–shell and shell–shell interfaces, even with a higher density than the bulk‐phase defects in inner core. Because of such higher density of interfacial defects, the kinetic energies transferred from energetic electrons to atoms may trigger the faster Na/F atom ejections and outward atom migrations in the coating layers than in the inner core of NPs, which ultimately results in the in situ formation of novel yolk–shell UCNPs. These findings provide new insights into interfacial defects in homogeneous core–shell structured NaLnF_4_ NPs, and pave the way toward utilizing the interactions of high‐energy particles with materials for in situ fabrication of novel nanostructures.

## 1 Introduction

Over the past decade, sodium lanthanide fluoride (NaLnF_4_) nanoparticles (NPs) with distinct downshifting and/or upconversion (UC) luminescence properties have attracted considerable attention due to their widespread applications in optics, biosensing, and drug delivery.^1^ Transmission electron microscopy (TEM) is usually utilized for the morphology and structure characterizations of NaLnF_4_ NPs based on various interactions of electron‐beam (e‐beam) with materials.^2^ However, the radiation damages induced by the e‐beam, such as localized voids formed in NPs or cracks appeared on the surfaces of NPs, are often observed during the characterizing process.^3^ Albeit deleterious, such radiation damages also offer opportunities for fabricating intriguing nanostructures. For instance, Feng et al. and Sun et al. reported the in situ nanoscale formation of hollow NaLnF_4_:Yb/Er UCNPs upon e‐beam irradiation.^2^ Their findings indicate that e‐beam irradiation bears unique advantages against traditional synthetic strategies in tailoring the nanostructures of NaLnF_4_ NPs.^4^

Homogeneous core–shell structured NaLnF_4_ NPs, in which an epitaxial shell (with closely matched lattice structure and composition to that of core) is grown on the core NP through a seed‐mediated method, are commonly adopted to construct inert shell to passivate surface photoluminescence (PL) quenchers or incorporate various lanthanide ions into separate shells to achieve distinct optical properties through the manipulation of intershell energy transfer.^5^ Generally, the passivation of surface PL quenchers includes two aspects: the spatial isolation of active lanthanide ions in core from the high‐energy vibrations of surface organic ligands by inert shell, and the elimination of original surface defects on core through shell coating.^6^

**Figure 1:**
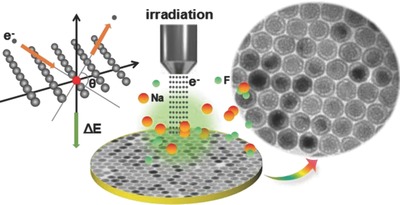
Schematic illustration of in situ fabrication of yolk–shell NaLuF_4_ UCNPs by e‐beam irradiation.

Although it is a consensus that homogeneous epitaxial shell growth is able to reduce the original surface defects on the as‐prepared core,[[qv: 6b,c]] we herein reveal that, for core–shell–shell NaLuF_4_:Gd/Yb/Er@NaLuF_4_:Nd/Yb@NaLuF_4_ UCNPs, considerable defects still exist at the core–shell and shell–shell interfaces with a higher density than the bulk‐phase defects in inner core. By utilizing these interfacial defects, novel yolk–shell NaLuF_4_ UCNPs with core‐void‐shell structure are fabricated in situ through e‐beam irradiation to the solid core–shell–shell UCNPs (**Figure** **1**).

## 2 Results and Discussion

**Figure 2:**
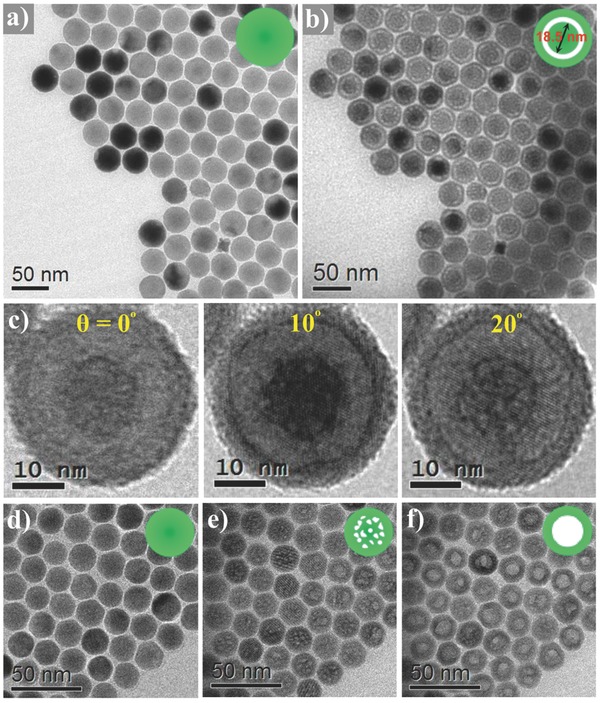
TEM images of core–shell–shell NPs a) before and b) after e‐beam irradiation for 30 s. c) TEM images of a yolk–shell NP with tilting angles from 0° to 20°. TEM images of core‐only NaLuF_4_:Gd/Yb/Er NPs d) before and after e) 60 and f) 90 s e‐beam irradiation.

Core–shell–shell NaLuF_4_:Gd/Yb/Er@NaLuF_4_:Nd/Yb@NaLuF_4_ UCNPs were synthesized through a seed‐mediated method,^7^ which involves the growth of core followed by the successive epitaxial deposition of shells. Typical low‐magnification TEM images of the NPs upon quick exposure to the e‐beam show that the core NaLuF_4_:Gd/Yb/Er, core–shell NaLuF_4_:Gd/Yb/Er@NaLuF_4_:Nd/Yb, and core–shell–shell NPs are nearly spherical with an average diameter of 19.0, 22.5, and 34.1 nm, respectively (**Figure** **2**a, and Figure S1, Supporting Information), as corroborated by the incrementally narrowed powder X‐ray diffraction peaks, which are well indexed as the hexagonal phase of NaLuF_4_ (Figure S2, Supporting Information). Moreover, the energy‐dispersive X‐ray (EDX) element mapping analysis for as‐prepared core–shell–shell NPs illustrates that the element distributions in NPs are consistent with the designed compositions, thus further confirming the core–shell–shell structure of NPs (Figure S3, Supporting Information). The remarkably enhanced UC emissions and prolonged luminescence lifetimes in core–shell–shell NPs upon 980 nm excitation as well as the strong UC emissions under 808 nm irradiation indicate that core–shell–shell strategy involving the doping of Nd^3+^ as sensitizer ion in the middle shell facilitates photon UC of Er^3+^ with Yb^3+^ acting as an energy transfer bridge, and the coating of outer inert shell enables surface passivation to avoid UC quenching (Figures S4–S6, Supporting Information).^8^

The core–shell–shell NPs were further observed by high‐magnification TEM images. Interestingly, yolk–shell NaLuF_4_ NPs (with an average inner core size of 18.5 nm) maintaining the original outer size were formed in situ under the e‐beam irradiation (with an acceleration voltage of 200 kV, and a current density of 40.0 A cm^−2^) upon the solid NPs assemblies for 30 s (Figure 2b and Movie S1, Supporting Information). It can be seen that the average size of retained inner core is nearly equal to that of pristine NaLuF_4_:Gd/Yb/Er core, while the previous middle NaLuF_4_:Nd/Yb shell and the retained inner NaLuF_4_:Gd/Yb/Er core are completely separated by the void for the vast majority of yolk–shell NPs. The selected‐area electron diffraction (SAED) patterns of the solid and yolk–shell NPs assemblies are compared in Figure S7 (Supporting Information). The identical SAED patterns reveal that the yolk–shell NPs retain the integrity of hexagonal crystal structure, which is also confirmed by the high‐resolution TEM (HRTEM) observation (Figure S8, Supporting Information). To further investigate the yolk–shell structure, TEM images were acquired through a tilting angle series from 0° to 20°. As shown in Figure 2c, the profile of the yolk–shell structure remains unchanged with tilting, indicating that the yolk–shell structure exhibits central symmetry. EDX analysis was also conducted to investigate the composition changes in NPs after the yolk–shell conversion (Figure S9, Supporting Information). By normalizing the atomic ratios of the elements with reference to that of Lu (Table S1, Supporting Information), it can be seen that both the contents of Na and F atoms are diminished greatly in the yolk–shell NPs in comparison to that in the solid counterparts, while the contents of Nd atoms (originally doped in the middle NaLuF_4_:Nd/Yb shell) and the other lanthanide (Ln) atoms remain nearly unchanged, which demonstrates that the Na and F atoms were selectively ejected out of NPs, that is, NaLnF_4_ units in crystal lattice were partially decomposed into lanthanide fluoride clusters LnF*_x_*. Similar solid‐to‐yolk‐shell conversion was observed in core–shell NaLuF_4_:Gd/Yb/Er@(NaLuF_4_:Nd/Yb or NaLuF_4_) UCNPs (Figure S10, Supporting Information). By contrast, for core‐only NaLuF_4_:Gd/Yb/Er counterparts (Figure 2d), no obvious changes emerged after 30 s irradiation, “bullet‐riddled” intermediates were captured after 60 s irradiation (Figure 2e), and a solid‐to‐hollow conversion was only observed after 90 s irradiation (Figure 2f). These phenomena indicate that core–shell structure is a prerequisite for the solid‐to‐yolk‐shell conversion.

**Figure 3:**
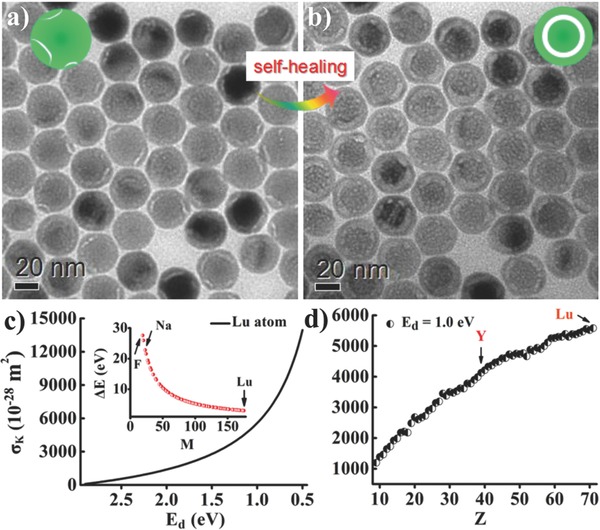
a) TEM image of core–shell–shell NPs after 30 s irradiation with a current density of 1.6 A cm^−2^. b) TEM image of NPs shown in (a) after an additional 30 s irradiation with a current density of 40 A cm^−2^. c) σ_K_ for Lu atom as a function of *E*_d_. The inset shows calculated Δ*E* for atoms with different M values. d) Calculated σ_K_ for atoms with different *Z* values by setting *E*_d_ = 1.0 eV.

Such unique yolk–shell conversion in core–shell–shell NPs depends critically on the current density and duration of e‐beam irradiation. Particularly, no yolk–shell conversion was observed under e‐beam irradiation with a current density of 1.6 A cm^−2^ for 30 s. Instead, fractures appeared on the surface of NPs (**Figure** **3**a). Unexpectedly, upon additional e‐beam irradiation to the fractured NPs with a current density of 40.0 A cm^−2^ for 30 s, we achieved the solid‐to‐yolk‐shell conversion, accompanied by the bridging and reconfiguration of the surface fractures (Figure 3b). Such self‐healing effect on surface fractures reveals that there exist atom migrations to repair the fractures during the e‐beam irradiation.[[qv: 3b]] Theoretically, the atom migrations under e‐beam irradiation are induced by the elastic scattering of energetic electrons by atoms.^9^ We propose an atom displacement model (Equations (1) and (2) in the Experimental Section) to depict the elastic scattering induced atom migration. During the elastic scattering, the kinetic energy Δ*E* transferred from beam electron to an atom can cause a lattice atom to become an interstitial atom or be ejected, which is usually defined as atom displacement, and collective atom displacements can induce directional migration of atoms.^9^, ^10^

According to Equations (1) and (2), the likelihood of atom displacement (denoted by the atom displacement cross section σ_K_) during the 200 keV e‐beam irradiation is dependent on three variables, *E*_d_ (the displacement threshold energy for an atom), *Z* (atomic number), *M* (atomic mass), and σ_K_ increases steeply with decreasing *E*_d_ (Figure 3c,d). Note that *E*_d_ is associated with the lattice energy.[[qv: 3b]] Theoretical calculations show that the lattice energy of NaF (910 kJ mol^−1^)^11^ is much lower than that of NaLuF_4_ (5111 kJ mol^−1^) or LuF_3_ (4968 kJ mol^−1^) (Equation (4) in the Experimental Section). Meanwhile, the Δ*E* values estimated for Na and F atoms are much larger than that for Lu atom (inset of Figure 3c). Thus, Na/F atoms in the NaLnF_4_ units are easily knocked out of NPs in view of its relatively weak binding strength of Na—F bond. In sharp contrast, the Ln atoms are difficult to be ejected out of NPs, due to the high binding energy for Ln—F bond. Nevertheless, the collective displacements of Ln/F atom powered by high Δ*E* can induce outward Ln/F atom migrations in NPs. Under such circumstances, the massive atom displacements evoked both Na/F atom ejections and outward atom migrations, which subsequently resulted in the formation of voids in NPs. As illustrated in Figure 2b and Movie S1 (Supporting Information), upon e‐beam irradiation to the core–shell–shell NPs, small voids were preferentially produced in the coating layers, and subsequently the voids coalesced to form a large spherical shell‐shaped void, giving rise to the solid‐to‐yolk‐shell conversion. It is reasonable to assume that the Na/F atom ejections and outward atom migrations in the coating layers are faster than that in the inner core upon e‐beam irradiation, which eventually leads to the solid‐to‐yolk‐shell conversion.

**Figure 4:**
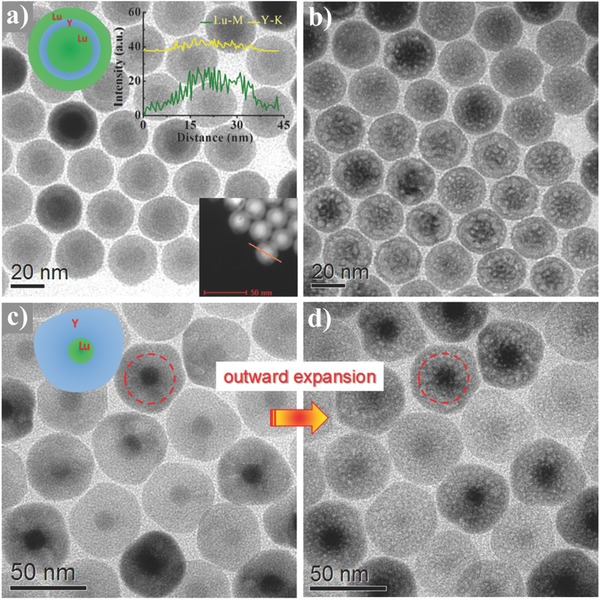
TEM images of NaLuF_4_:Gd/Yb/Er@NaYF_4_:Nd/Yb@NaLuF_4_ NPs a) before and b) after e‐beam irradiation for 120 s. The bottom and top insets of (a) show the high‐angle annular dark‐field scanning transmission electron microscopy (HAADF‐STEM) image and EDX line scan, respectively. TEM images of NaLuF_4_:Gd/Yb/Er@NaYF_4_:Nd/Yb NPs c) before and d) after e‐beam irradiation for 120 s.

To verify the above conversion mechanism, NaLuF_4_:Gd/Yb/Er@NaYF_4_:Nd/Yb@NaLuF_4_ NPs as control were synthesized under the otherwise same conditions as that of NaLuF_4_:Gd/Yb/Er@NaLuF_4_:Nd/Yb@NaLuF_4_ NPs except for varied middle‐shell host components, and exposed to e‐beam irradiation as well (**Figure** **4**a and its inset). It is well known that NaYF_4_ is isostructural and has analogous lattice energy and chemical properties to NaLuF_4_,^12^ except for the much smaller *Z* and *M* for Y than for Lu. According to the atom displacement model (Figure 3d), the σ_K_ for Y is much smaller than that for Lu. As such, no detectable change of morphology was observed after 30 s irradiation, whereas only part of the irradiated NPs experienced solid‐to‐yolk‐shell conversion after a prolonged irradiation of 120 s (Figure S11, Supporting Information, and Figure 4b), which is much longer and less effective as compared to the case of NaLuF_4_:Gd/Yb/Er@NaLuF_4_:Nd/Yb@NaLuF_4_ NPs. It can be well interpreted since the speeds of Na/F atom ejections and outward atom migrations in the coating layers are relatively reduced due to the smaller σ_K_ for Y. Moreover, thick NaYF_4_:Nd/Yb shell was adopted to deposit on core NaLuF_4_:Gd/Yb/Er NP (Figure 4c) to further reduce the speeds of Na/F atom ejections and outward atom migrations in the coating layers. As expected, instead of solid‐to‐yolk‐shell conversion, a volume expansion of inner NaLuF_4_:Gd/Yb/Er core into NaYF_4_:Nd/Yb shell (as marked by the dashed rings in Figure 4c,d) induced by the outward atom migrations was clearly observed. These observations strongly support our assumption that the solid‐to‐yolk‐shell conversion is caused by the differentiated speeds of Na/F atom ejections and outward atom migrations in NPs.

Core‐only NaLuF_4_:Gd/Yb/Er NPs, unlike their core–shell–shell counterparts, behave very differently upon e‐beam irradiation. Small voids uniformly distributed across the whole NP first appeared to form the “bullet‐riddled” intermediates (Figure 2e), then the voids merged together to form a large hollow void, exhibiting the solid‐to‐hollow conversion. To unravel the underlying mechanism for the above difference between core‐only and core–shell–shell NPs, we rationalize that, for core–shell–shell NPs, there exist core–shell and shell–shell interfacial defects with a higher density than bulk‐phase defects in inner core, which results in the lower average *E*_d_ for atoms in the coating layers of NPs. As illustrated in Figure 3c, σ_K_ increases rapidly with decreasing *E*_d_. As such, the larger σ_K_ for atoms allows for the rather faster Na/F atom ejections and outward atom migrations in the coating layers than that in the inner core upon e‐beam irradiation. As a result, yolk–shell nanostructure is formed (Figure S12, Supporting Information). While for the core‐only NaLuF_4_:Gd/Yb/Er NPs, undifferentiated speeds of Na/F atom ejections and outward atom migrations, dictated by the relatively uniformly distributed defects in NPs, are responsible for the formation of hollow structures (Figure 2f).

**Figure 5:**
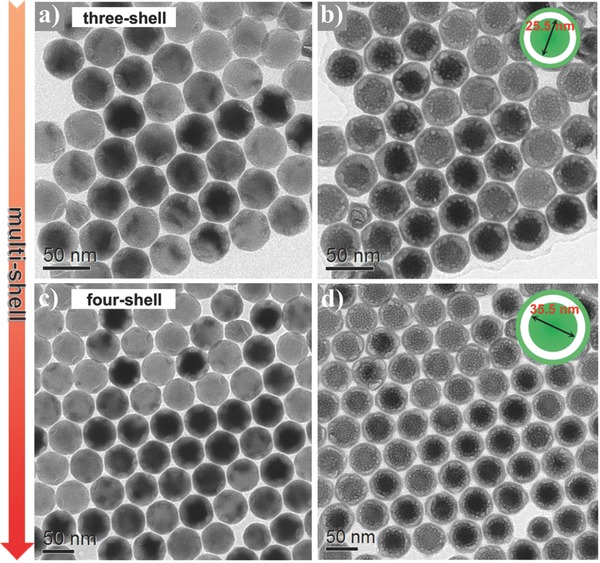
TEM images of three‐shell NaLuF_4_ UCNPs (42.7 nm) before a) and after b) e‐beam irradiation for 30 s. TEM images of four‐shell NaLuF_4_ UCNPs (51.9 nm) before c) and after d) e‐beam irradiation for 30 s.

For homogeneous core–shell structured NaLnF_4_ NPs, it is generally accepted that, the core–shell interface might be defect‐free, because the coating of epitaxial shell is deemed to play a key role for healing original surface defects on the core.[[qv: 6c]] Oppositely, during the epitaxial deposition of shell on core, lattice stress is unavoidable due to the complicated 3D geometry of the growth surface, and lattice stress can be relieved by the formation of crystal defects at the core–shell interface.^13^ As a result of the existence of such interfacial defects, core–shell structured NaYF_4_ or NaGdF_4_ (isostructural to NaLuF_4_) NPs also experienced the solid‐to‐yolk‐shell conversion analogous to core–shell structured NaLuF_4_ NPs upon e‐beam irradiation (Figure S13, Supporting Information). Although interfacial defects are ubiquitous in core–shell structured NaLnF_4_ NPs, they can be minimized by postannealing.^14^ To exemplify this, the as‐prepared core–shell–shell NPs were irradiated with fs laser pulses (800 nm, 1 kHz) after deposition on the TEM grid. As shown in Figure S14a (Supporting Information), the core–shell–shell NPs merged with each other and were recrystallized to form larger irregularly shaped NPs while retaining their original hexagonal crystal structure. Upon e‐beam irradiation to these laser‐annealed NPs, no radiation damages were observed within the time scale of 30 s, while only “bullet‐riddled” or hollow NPs appeared after a long irradiation time of 120 s (Figure S14b, Supporting Information). These phenomena demonstrate that the original interfacial defects in NPs were largely reduced by the pulsed laser annealing.[[qv: 14b,15]] Besides laser annealing, thermal annealing is more prevailingly used to minimize crystal defects.^16^ This can be fulfilled by successively coating additional NaLuF_4_ shells on the core–shell–shell NaLuF_4_:Gd/Yb/Er@NaLuF_4_:Nd/Yb@NaLuF_4_ NPs, during which the previous core–shell–shell NPs should experience a postgrowth annealing process (i.e., recrystallization).^17^ That is, the original interfacial defects in core–shell–shell NPs may be gradually diminished through repeated recrystallizations during the coating of additional shells on core–shell–shell NPs. Actually, when e‐beam irradiation was conducted on three‐shell (i.e., NaLuF_4_:Gd/Yb/Er@NaLuF_4_:Nd/Yb@NaLuF_4_@NaLuF_4_) and four‐shell NaLuF_4_ UCNPs, yolk–shell NPs were produced in situ with the size of retained inner core increasing from 18.5 to 25.5 and 35.5 nm, respectively (**Figure** **5**a–d), as compared to the case of core–shell–shell UCNPs (Figure 2b). It is worthy of mentioning that the average inner core size (35.5 nm) of yolk–shell NPs converted from four‐shell NPs is slightly larger than the average size of previous core–shell–shell NPs (34.1 nm), which indicates that the whole core–shell–shell NP is retained in the yolk–shell NP owing to the gradual reduction of interfacial defects in core–shell–shell NP. Therefore, through direct manipulation of the gradient distribution of interfacial defects in NPs by multishell coating techniques, we are able to finely fabricate in situ yolk–shell NaLuF_4_ UCNPs with tunable inner core size under e‐beam irradiation.

## 3 Conclusion

In summary, we have achieved in situ solid‐to‐yolk‐shell structure conversion in homogeneous core–shell structured NaLuF_4_ UCNPs, which was induced by the differentiated speeds of Na/F atom ejections and outward atom migrations in NPs under e‐beam irradiation. We revealed that the in situ formation of such novel yolk–shell UCNPs is dictated by the ubiquitous interfacial defects at the core–shell or shell–shell interfaces of core–shell structured NPs. The findings offer new understanding for the role of the previously unnoticed interfacial defects in homogeneous core–shell structured NPs. More importantly, by controlling the gradient distribution of interfacial defects in core–shell structured NPs through multishell coating, we successfully fabricated yolk–shell NaLuF_4_ UCNPs with size‐tunable inner core by e‐beam irradiation, which is of methodological significance for the utilization of high‐energy particle radiation to in situ fabricate functionalized NPs with novel structural features that are highly promising building blocks for nanodevice applications.

## 4 Experimental Section

*Chemicals and Materials*: Ln_2_O_3_ (Ln = Lu, Gd, Yb, Er, Nd, Y) (99.99%), trifluoroacetic acid, cyclohexane, and ethanol were purchased from Sinopharm Chemical Reagent Co., China. Oleic acid (OA), oleylamine (OM), and 1‐octadecence (ODE) were purchased from Sigma‐Aldrich (China). All the chemical reagents were used as received without further purification.

*Synthesis of Core NaLuF_4_:Gd/Yb/Er NPs*: NaLuF_4_:Gd/Yb/Er were synthesized via a modified thermal decomposition process.^18^ In a typical process, Ln(CF_3_COO)_3_ (0.5 mmol, 45% mol of Lu, 33% mol of Gd, 20% mol of Yb, 2% mol of Er) and Na(CF_3_COO) (0.75 mmol) were added to a 50 mL flask containing OA (5.9356 g), OM (2.9627 g), and ODE (3.0197 g) at room temperature (RT). The obtained mixture was heated at 120 °C for 15 min under magnetic stirring in N_2_ atmosphere to dissolve the trifluoroacetate precursors and remove the residual water and oxygen. Subsequently, the resulting transparent solution was heated to 310 °C under N_2_ flow with vigorous stirring for 60 min, and then cooled to RT naturally. The resulting NaLuF_4_:Gd/Yb/Er NPs were precipitated by addition of ethanol, collected via centrifugation, washed with ethanol for two times, and redispersed in cyclohexane.

*Synthesis of Core–Shell NaLuF_4_:Gd/Yb/Er@NaLuF_4_:Nd/Yb NPs, Core–Shell NaLuF_4_:Gd/Yb/Er@NaLuF_4_ NPs, Core–Shell NaLuF_4_:Gd/Yb/Er@NaYF_4_:Nd/Yb NPs, and Thick Shell NaLuF_4_:Gd/Yb/Er@NaYF_4_:Nd/Yb NPs*: The core–shell structured NPs were fabricated using the seed‐mediated method. In a typical synthetic procedure for core–shell NaLuF_4_:Gd/Yb/Er@NaLuF_4_:Nd/Yb NPs, NaLuF_4_:Gd/Yb/Er NPs were added to a 50 mL flask with OA (6.2954 g) and ODE (5.5626 g). The solution was stirred at 90 °C in N_2_ atmosphere to remove cyclohexane. The reaction mixture was then cooled to RT. Thereafter, Ln(CF_3_COO)_3_ (0.425 mmol, 80% mol of Lu, 10% mol of Nd, 10% mol of Yb) and Na(CF_3_COO) (0.425 mmol) were added. The mixture was heated at 120 °C for 15 min under magnetic stirring in N_2_ atmosphere to dissolve the trifluoroacetate precursors and remove the residual water and oxygen. Subsequently, the resulting transparent solution was heated to 300 °C under N_2_ flow with vigorous stirring for 60 min, and then cooled to RT naturally. The resulting NaLuF_4_:Gd/Yb/Er@NaLuF_4_:Nd/Yb NPs were precipitated by addition of ethanol, collected via centrifugation, washed with ethanol for two times, and redispersed in cyclohexane.

The synthetic procedures for core–shell NaLuF_4_:Gd/Yb/Er@NaLuF_4_ NPs, core–shell NaLuF_4_:Gd/Yb/Er@NaYF_4_:Nd/Yb NPs, and thick shell NaLuF_4_:Gd/Yb/Er@NaYF_4_:Nd/Yb NPs were identical to that for core–shell NaLuF_4_:Gd/Yb/Er@NaLuF_4_:Nd/Yb NPs, except for that 1 mmol of Lu(CF_3_COO)_3_ and 1 mmol of Na(CF_3_COO) were added as shell precursors, and the reaction time for shell coating is 120 min for core–shell NaLuF_4_:Gd/Yb/Er@NaLuF_4_ NPs; 0.425 mmol of Ln(CF_3_COO)_3_ (80% mol of Y, 10% mol of Nd, 10% mol of Yb) and 0.425 mmol of Na(CF_3_COO) were added as shell precursors for core–shell NaLuF_4_:Gd/Yb/Er@NaYF_4_:Nd/Yb NPs; and 3 mmol of Ln(CF_3_COO)_3_ (80% mol of Y, 10% mol of Nd, 10% mol of Yb) and 3 mmol of Na(CF_3_COO) were added as shell precursors, and the reaction time for shell coating is 150 min for thick shell NaLuF_4_:Gd/Yb/Er@NaYF_4_:Nd/Yb NPs.

*Synthesis of Core–Shell–Shell NaLuF_4_:Gd/Yb/Er@NaLuF_4_:Nd/Yb@NaLuF_4_ NPs and Core–Shell–Shell NaLuF_4_:Gd/Yb/Er@NaYF_4_:Nd/Yb@NaLuF_4_ NPs*: The core–shell–shell NPs were fabricated by using the presynthesized core–shell NPs as seeds. In a typical synthesis process for core–shell–shell NaLuF_4_:Gd/Yb/Er@NaLuF_4_:Nd/Yb@NaLuF_4_ NPs, NaLuF_4_:Gd/Yb/Er@NaLuF_4_:Nd/Yb NPs were added to a 50 mL flask with OA (6.2954 g) and ODE (5.5626 g). The solution was stirred at 90 °C in N_2_ atmosphere to remove cyclohexane. The reaction mixture was then cooled to RT. Thereafter, Lu(CF_3_COO)_3_ (0.5 mmol) and Na(CF_3_COO) (0.5 mmol) were added. The mixture was heated at 120 °C for 15 min under magnetic stirring in N_2_ atmosphere to dissolve the trifluoroacetate precursors and remove the residual water and oxygen. Subsequently, the resulting transparent solution was heated to 300 °C under N_2_ flow with vigorous stirring for 60 min, and then cooled to RT naturally. The resulting NaLuF_4_:Gd/Yb/Er@NaLuF_4_:Nd/Yb@NaLuF_4_ NPs were precipitated by addition of ethanol, collected via centrifugation, washed with ethanol for two times, and redispersed in cyclohexane.

The synthetic procedure for core–shell–shell NaLuF_4_:Gd/Yb/Er@NaYF_4_:Nd/Yb@NaLuF_4_ NPs was identical to that for core–shell–shell NaLuF_4_:Gd/Yb/Er@NaLuF_4_:Nd/Yb@NaLuF_4_ NPs, except for that the presynthesized core–shell NaLuF_4_:Gd/Yb/Er@NaYF_4_:Nd/Yb NPs were added as seeds for core–shell–shell NaLuF_4_:Gd/Yb/Er@NaYF_4_:Nd/Yb@NaLuF_4_ NPs.

*Synthesis of Three‐Shell NaLuF_4_:Gd/Yb/Er@NaLuF_4_:Nd/Yb@NaLuF_4_@NaLuF_4_ NPs*: The three‐shell NaLuF_4_:Gd/Yb/Er@NaLuF_4_:Nd/Yb@NaLuF_4_@NaLuF_4_ NPs were fabricated by using the presynthesized core–shell–shell NaLuF_4_:Gd/Yb/Er@NaLuF_4_:Nd/Yb@NaLuF_4_ NPs as seeds. The synthetic procedure for coating the third NaLuF_4_ shell was the same as the aforementioned synthesis of core–shell–shell NaLuF_4_:Gd/Yb/Er@NaLuF_4_:Nd/Yb@NaLuF_4_ NPs.

*Synthesis of Four–Shell NaLuF_4_:Gd/Yb/Er@NaLuF_4_:Nd/Yb@NaLuF_4_@NaLuF_4_@NaLuF_4_ NPs*: The four‐shell NaLuF_4_:Gd/Yb/Er@NaLuF_4_:Nd/Yb@NaLuF_4_@NaLuF_4_@NaLuF_4_ NPs were fabricated by using the presynthesized three‐shell NaLuF_4_:Gd/Yb/Er@NaLuF_4_:Nd/Yb@NaLuF_4_@NaLuF_4_ NPs as seeds. The synthetic procedure for coating the fourth NaLuF_4_ shell was the same as the aforementioned synthesis of core–shell–shell NaLuF_4_:Gd/Yb/Er@NaLuF_4_:Nd/Yb@NaLuF_4_ NPs.

*Synthesis of Core–Shell NaYF_4_:Gd/Yb/Er@NaYF_4_:Nd/Yb and NaGdF_4_:Yb/Er@NaGdF_4_:Nd/Yb NPs*: The synthetic procedures for core–shell NaYF_4_:Gd/Yb/Er@NaYF_4_:Nd/Yb and NaGdF_4_:Yb/Er@NaGdF_4_:Nd/Yb NPs were identical to that for core–shell NaLuF_4_:Gd/Yb/Er@NaLuF_4_:Nd/Yb NPs, except for that the added Lu(CF_3_COO)_3_ precursor was substituted by Y(CF_3_COO)_3_ and Gd(CF_3_COO)_3_, respectively.

*Structural and Optical Characterization*: Powder X‐ray diffraction (XRD) patterns of the samples were collected on an X‐ray diffractometer (MiniFlex2, Rigaku) with Cu Kα1 radiation (λ = 0.154187 nm). TEM measurements were performed on a JEM‐2010 TEM with an acceleration voltage of 200 KV. e‐beam irradiation was carried out on a JEM‐2010 TEM with an acceleration voltage of 200 kV and a current density of 40.0 or 1.6 A cm^−2^. High‐angle annular dark‐field scanning transmission electron microscopy (HAADF‐STEM) image and EDX line scan and element mapping, as well as the dynamic process of solid‐to‐yolk‐shell structure conversion in single core–shell–shell NaLuF_4_:Gd/Yb/Er@NaLuF_4_:Nd/Yb@NaLuF_4_ NP upon e‐beam irradiation were captured using a FEI Tecnai F20 TEM operating at an acceleration voltage of 200 kV (the capture of dynamic process of yolk–shell conversion was conducted with a current density of 40.0 A cm^−2^). The as‐prepared core–shell–shell NPs deposited on the TEM grid were irradiated with fs laser pulses generated by a regeneratively amplified fs Ti:sapphire laser system (800 nm, 1 kHz, Spitfire Pro‐F1KXP, Spectra‐Physics), which was seeded by a fs Ti‐sapphire oscillator (80 MHz, 710–920 nm, Maitai XF‐1, Spectra‐Physics). Upconversion luminescence (UCL) spectra were collected under 980 or 808 nm laser excitation provided by corresponding continuous‐wave laser diode. UCL lifetimes were measured with a customized ultraviolet (UV) to mid‐infrared steady‐state and phosphorescence lifetime spectrometer (FSP920‐C, Edinburgh) equipped with a digital oscilloscope (TDS3052B, Tektronix) and a tunable mid‐band optical parametric oscillator (OPO) pulse laser as the excitation source (410–2400 nm, 10 Hz, pulse width of ≈5 ns, Vibrant 355II, OPOTEK). All the spectral data collected were corrected for the spectral response of the spectrometer.

*Atom Displacement Model*: Theoretically, the atom migrations under e‐beam irradiation are attributed to the interactions between energetic electrons and atoms, that is, elastic scattering of beam electron by atomic nuclei and inelastic scattering by atomic electrons. The geometry of elastic scattering of a beam electron by an atomic nuclei is shown below.


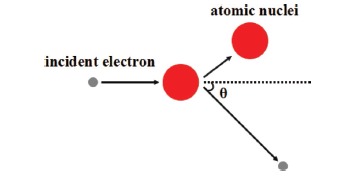


For elastic scattering, conservation of energy and momentum dictates that an electron deflected through an angle θ in the Coulomb field of atomic nuclei (atomic mass *M*) must transfer a certain amount of energy to an atom. The transferred kinetic energy is given by^10^

(1) $$\Delta E=\frac{2(E+2E_{0})E}{Mc^{2}}\cdot \sin^{2}\frac{\theta }{2}$$

where *E* is the kinetic energy of beam electron, *E*_0_ = *m*_0_ *c* ^2^ (*m*_0_ is the rest mass of electron), and *c* is the speed of light in vacuum. It is worth noting that the transferred kinetic energy Δ*E* can cause a lattice atom to become an interstitial atom or be ejected, which is usually defined as atom displacement, and massive collective atom displacements can induce directional migration of atoms. Moreover, the likelihood of atom displacement (denoted by the atom displacement cross section σ_K_) during the 200 keV e‐beam irradiation can be written in the following analytical form^10^

(2) $$\sigma_{K}=0.\mathrm{5541}\cdot \frac{Z^{2}(T_{m}-E_{d})}{\left(1+\alpha )(\alpha T_{m}+E_{d}\right)}\approx 0.\mathrm{5541}\cdot Z^{2}\left(\frac{T_{m}}{E_{d}}-1\right)$$

where *T*_m_ = 2(*E* + 2*E*_0_)*E*/(*Mc* ^2^), *Z* is the atomic number, α is a screening factor (*α* **≪** 1), and *j* is the current density of e‐beam. In contrast to elastic scattering, inelastic scattering converts part of kinetic energy of beam electron into atomic electron excitations, which subsequently causes bond breakage, heating effect, photon emission, secondary/Auger electrons, etc. For light elements, inelastic scattering is dominant, while elastic scattering is prevailing for heavy atoms. The ratios of the total inelastic to total elastic cross section (σ_inelastic_/σ_elastic_) for different elements can be calculated from^10^

(3) $$\begin{array}{c} \sigma_{\mathrm{inelastic}}/\sigma_{\mathrm{elastic}}=\frac{2}{Z}\ln\left[\frac{1194}{Z^{4/3}}E(1+E\mathrm{/1022})\right] \end{array}$$

where *Z* is atomic number, and *E* (the kinetic energy of the beam electron) is given in keV (*E* = 200). Through calculations, the σ_inelastic_/σ_elastic_ is 2.14 for F, 1.70 for Na, 0.39 for Y, 0.22 for Gd, and 0.20 for Lu, respectively. In theory, elastic scattering directly incurs atom displacements, while inelastic scattering can accelerate atom displacements by the inelastic scattering effects, such as bond breakage and heating effect, both of which reduce *E*_d_ to some extent.^9^ Moreover, under e‐beam irradiation with a given current density of *j*, the displacement rate *P* of an atom is determined by the equation *P* = σ_K_ ∙ *j*,^9^ namely, atom displacement is current density dependent. Hence, under e‐beam irradiation with low current density (such as 1.6 A cm^−2^), locally triggered minor atom displacements in NPs merely induced lattice stress to produce surface fractures instead of forming yolk–shell or hollow structures. Oppositely, during e‐beam irradiation with high current density (e.g., 40 A cm^−2^), massive atom displacements led to vast Na/F atom ejections and outward atom migrations, and simultaneously the faster Na/F ejections and atom migrations in the coating layers than in the inner core of core–shell–shell NPs resulted in the in situ formation of yolk–shell nanostructures. Besides, it should be noted that the sample thickness along the e‐beam direction is ≤*d* (*d* is the diameter of NP), thus the attenuation of e‐beam at depth is reasonably neglected for the simplicity of theoretical analysis.

*Calculations of Lattice Energies for Complex Ionic Solids*: The lattice energy (*U*_pot_) for an ionic crystal can be expressed by^11^

(4) $$\begin{array}{c} U_{\mathrm{pot}}=2I(\alpha V_{m}^{-\mathrm{1/3}}+\beta ) \end{array}$$

where *I* is an ionic strength related term (*I* = 1/2∑*n_i_Z_i_* ^2^, *n_i_* is the number of ions of type *i* per formula unit, and *Z_i_* represents the valence of *i* type ion), α (138.7 kJ mol^−1^ nm) and β (27.6 kJ mol^−1^) are empirical constants. According to Equation (4), *U*_pot_ is calculated to be 5111 kJ mol^−1^ for hexagonal NaLuF_4_ (JCPDS 27‐0726), 4968 kJ mol^−1^ for orthorhombic LuF_3_ (JCPDS 17‐0796), 5046 kJ mol^−1^ for hexagonal NaYF_4_ (JCPDS 16‐0334), and 4914 kJ mol^−1^ for orthorhombic YF_3_ (JCPDS 32‐1431), respectively.

## Conflict of Interest

The authors declare no conflict of interest.

## Supporting information

Supplementary

Supplementary
