# Supplementary material for: Interfacial Defects Dictated In Situ Fabrication of Yolk–Shell Upconversion Nanoparticles by Electron‐Beam Irradiation
Source: Adv Sci (Weinh). 2018 Jul 25;5(10):1800766. doi: 10.1002/advs.201800766 (PMC6193145; doi:10.1002/advs.201800766)
Supplement: Supplementary file 1 — Supplementary [file ADVS-5-1800766-s002.pdf]

Copyright WILEY-VCH Verlag GmbH & Co. KGaA, 69469 Weinheim, Germany, 2018.

Supporting Information

for *Adv. Sci.*, DOI: 10.1002/advs.201800766

Interfacial Defects Dictated In Situ Fabrication of Yolk–Shell Upconversion Nanoparticles by Electron-Beam Irradiation

Jin Xu, Datao Tu, Wei Zheng, Xiaoying Shang, Ping Huang, Yao Cheng, Yuansheng Wang, and Xueyuan Chen**

Supporting Information

Interfacial Defects Dictated In Situ Fabrication of Yolk-Shell Upconversion Nanoparticles by Electron-Beam Irradiation

Jin Xu, Datao Tu, Wei Zheng, Xiaoying Shang, Ping Huang, Yao Cheng,* Yuansheng Wang, and Xueyuan Chen*

Supplementary Table

Table S1. Elemental analyses of core-shell-shell $NaLuF_4$:Gd/Yb/Er@$NaLuF_4$:Nd/Yb@$NaLuF_4$ NPs before and after the solid-to-yolk-shell structure conversion.

Element	Atomic Mass	Atomic Ratio [a)]	
		Before	After
**F**	19	**4.363**	**2.889**
**Na**	23	**1.161**	**0.387**
Nd	144.2	0.057	0.050
Gd	157.3	0.096	0.114
Er	167.3	0.039	0.033
Yb	173	0.093	0.101
Lu	175	1.000	1.000

a) Normalized with reference to that of Lu.

Supplementary Movie

Movie S1. The dynamic process of solid-to-yolk-shell structure conversion in single core-shell-shell $NaLuF_4$:Gd/Yb/Er@$NaLuF_4$:Nd/Yb@$NaLuF_4$ NP upon e-beam irradiation.

Supplementary Figures

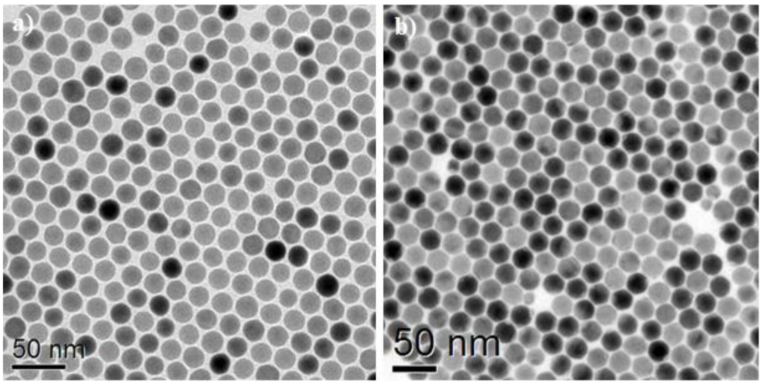

Figure S1. TEM images of a) core $NaLuF_4$:Gd/Yb/Er (19.0 nm) and b) core-shell $NaLuF_4$:Gd/Yb/Er@$NaLuF_4$:Nd/Yb (22.5 nm) NPs.

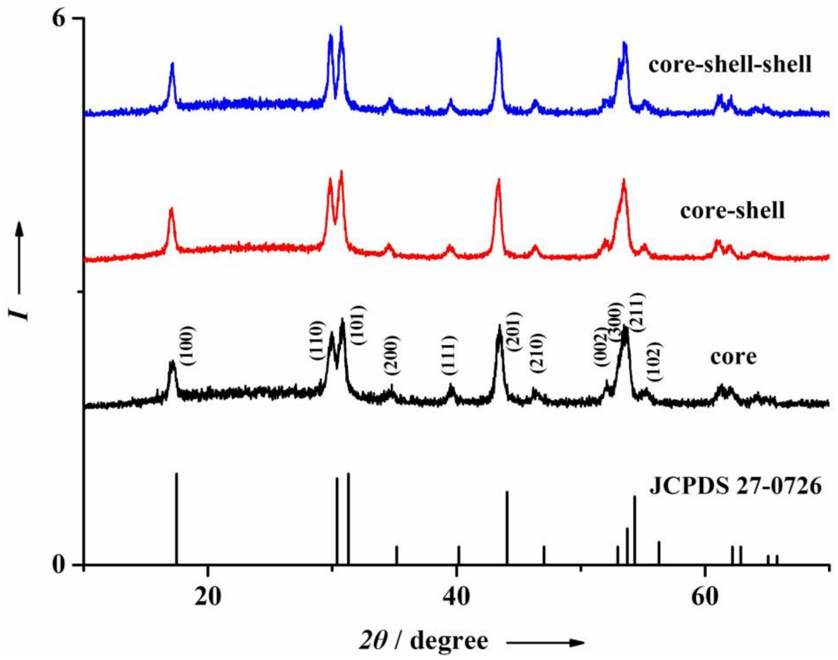

Figure S2. XRD patterns of core $NaLuF_4$:Gd/Yb/Er, core-shell $NaLuF_4$:Gd/Yb/Er@$NaLuF_4$:Nd/Yb, and core-shell-shell $NaLuF_4$:Gd/Yb/Er@$NaLuF_4$:Nd/Yb@$NaLuF_4$ NPs. All diffraction peaks match well with the standard pattern of hexagonal phase of $NaLuF_4$ (JCPDS No. 27-0726).

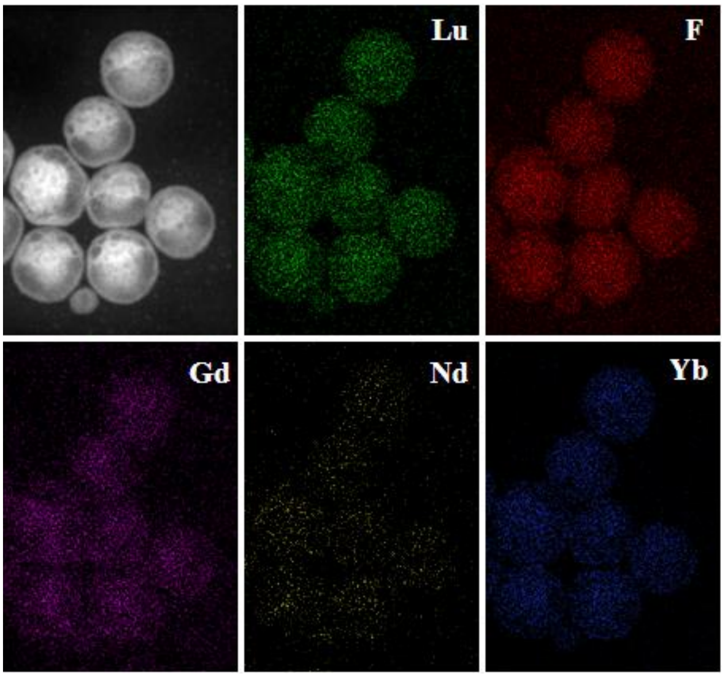

Figure S3. High-angle annular dark-field scanning transmission electron microscopy (HAADF-STEM) image and corresponding element mappings of Lu, F, Gd, Nd and Yb for as-prepared core-shell-shell $NaLuF_4$:Gd/Yb/Er@$NaLuF_4$:Nd/Yb@$NaLuF_4$ NPs.

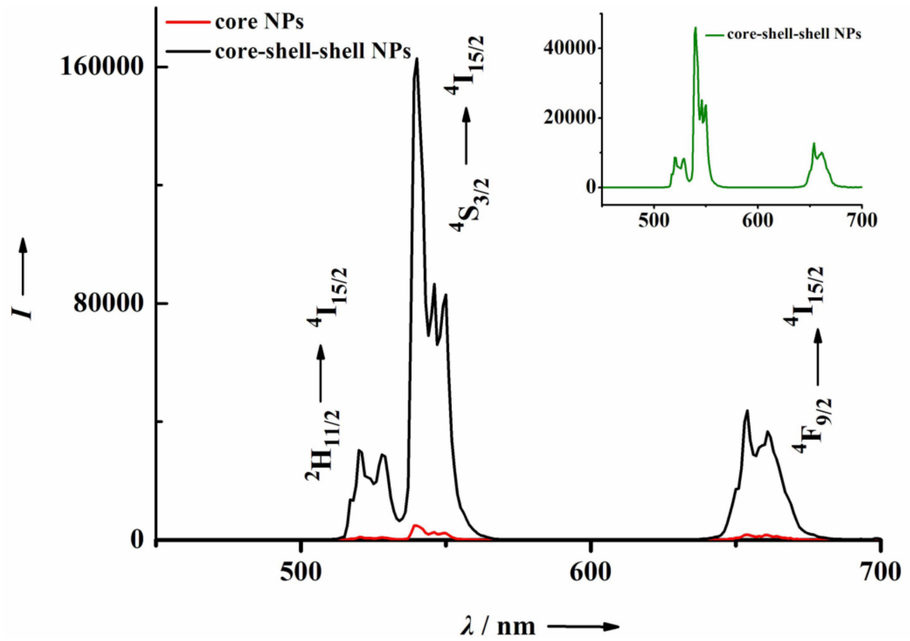

Figure S4. UCL spectra of core $NaLuF_4$:Gd/Yb/Er and core-shell-shell $NaLuF_4$:Gd/Yb/Er@$NaLuF_4$:Nd/Yb@$NaLuF_4$ NPs under 980 nm laser irradiation at a power density of about 3 W/cm^2. The inset shows the UCL spectrum of the core-shell-shell $NaLuF_4$:Gd/Yb/Er@$NaLuF_4$:Nd/Yb@$NaLuF_4$ NPs upon 808 nm laser excitation with a power density of ~4 W/cm^2.

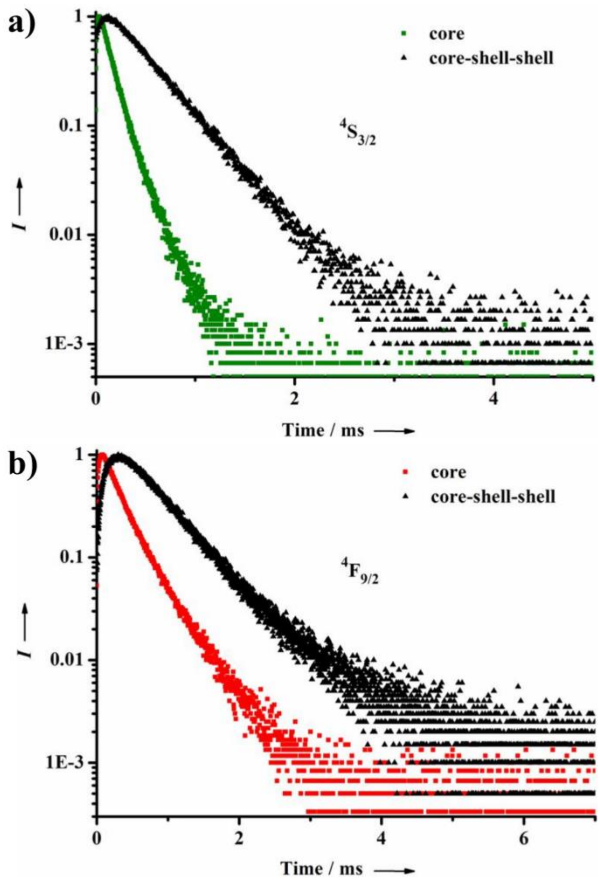

Figure S5. UCL decays from a) $^4S_{3/2}$ and b) $^4F_{9/2}$ of Er^{3+} in core $NaLuF_4$:Gd/Yb/Er and core-shell-shell $NaLuF_4$:Gd/Yb/Er@$NaLuF_4$:Nd/Yb@$NaLuF_4$ NPs. The UCL lifetime was determined by single-exponential fit to the decay. The UCL lifetimes of $^4S_{3/2}$ ($^4F_{9/2}$) were determined to be 0.13 (0.28) and 0.42 (0.59) ms for core and core-shell-shell NPs, respectively.

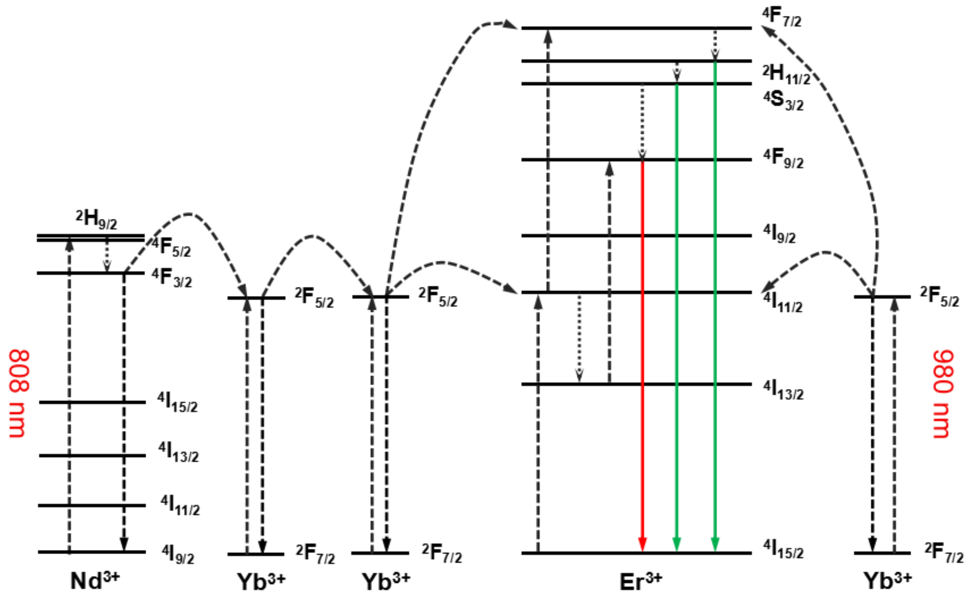

Figure S6. Energy level diagrams of Nd^{3+}, Yb^{3+} and Er^{3+} ions and the proposed energy transfer (ET) pathway for UC processes in the core-shell-shell $NaLuF_4$:Gd/Yb/Er@$NaLuF_4$:Nd/Yb@$NaLuF_4$ NPs under 808/980 nm excitation. The 808 nm laser firstly excites Nd^{3+} (doped in shell) to its $^4F_{5/2}$ state, which is followed by the nonradiative relaxation to the $^4F_{3/2}$ state. Subsequently, the energy transfers from the $^4F_{3/2}$ (Nd^{3+}) to the $^2F_{5/2}$ (Yb^{3+}), and then the typical UC in Er^{3+}-doped core is initiated through the successive $Yb^{3+}\rightarrow Yb^{3+}\rightarrow Er^{3+}$ ET processes. By contrast, for the case of 980 nm excitation, Yb^{3+} is directly excited to its $^2F_{5/2}$ state, and then the $Yb^{3+}\rightarrow Er^{3+}$ ET process initiates the UC in Er^{3+}-doped core.

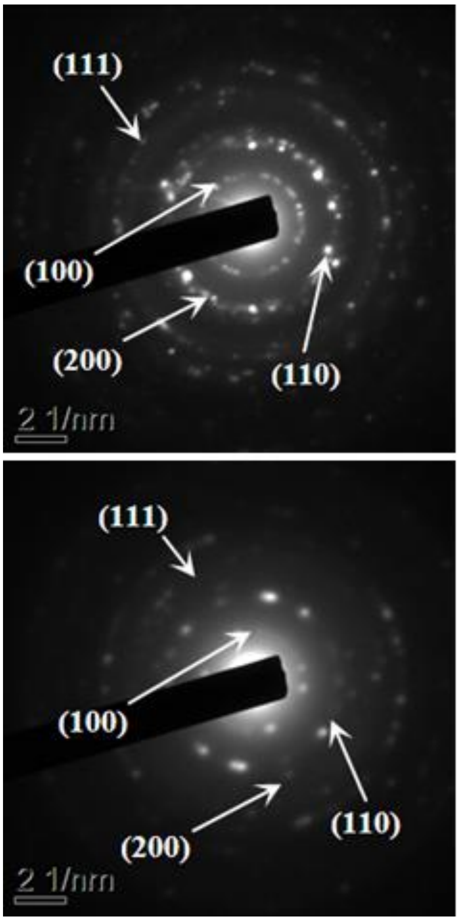

Figure S7. Selected-area electron diffraction (SAED) patterns of the solid core-shell-shell $NaLuF_4$:Gd/Yb/Er@$NaLuF_4$:Nd/Yb@$NaLuF_4$ (upper) and yolk-shell (lower) NPs assemblies.

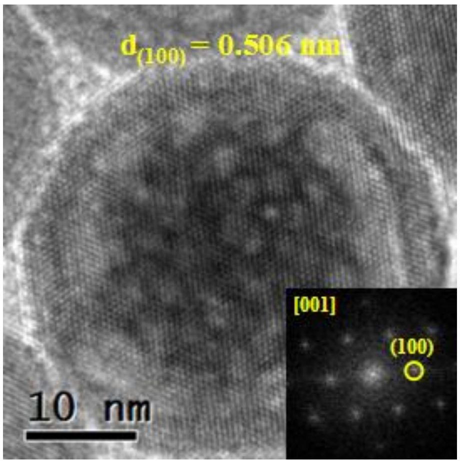

Figure S8. High resolution TEM (HRTEM) image of single yolk-shell NP and its corresponding fast Fourier transform (FFT) pattern (bottom inset).

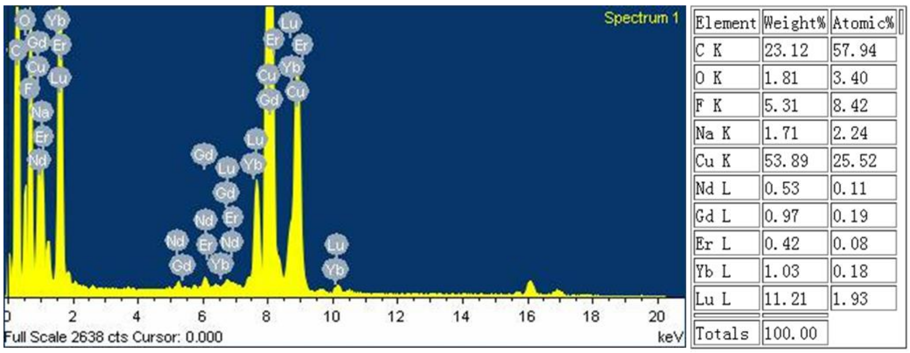

Element	Weight%	Atomic%
C K	23.12	57.94
O K	1.81	3.40
F K	5.31	8.42
Na K	1.71	2.24
Cu K	53.89	25.52
Nd L	0.53	0.11
Gd L	0.97	0.19
Er L	0.42	0.08
Yb L	1.03	0.18
Lu L	11.21	1.93
Totals	100.00	

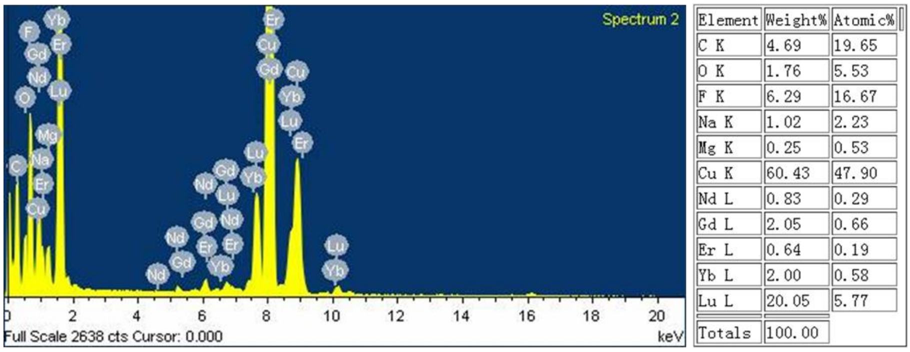

Element	Weight%	Atomic%
C K	4.69	19.65
O K	1.76	5.53
F K	6.29	16.67
Na K	1.02	2.23
Mg K	0.25	0.53
Cu K	60.43	47.90
Nd L	0.83	0.29
Gd L	2.05	0.66
Er L	0.64	0.19
Yb L	2.00	0.58
Lu L	20.05	5.77
Totals	100.00	

Figure S9. EDX spectroscopy analyses of core-shell-shell $NaLuF_4$:Gd/Yb/Er@$NaLuF_4$:Nd/Yb@$NaLuF_4$ NPs before (upper) and after (lower) the solid-to-yolk-shell structure conversion.

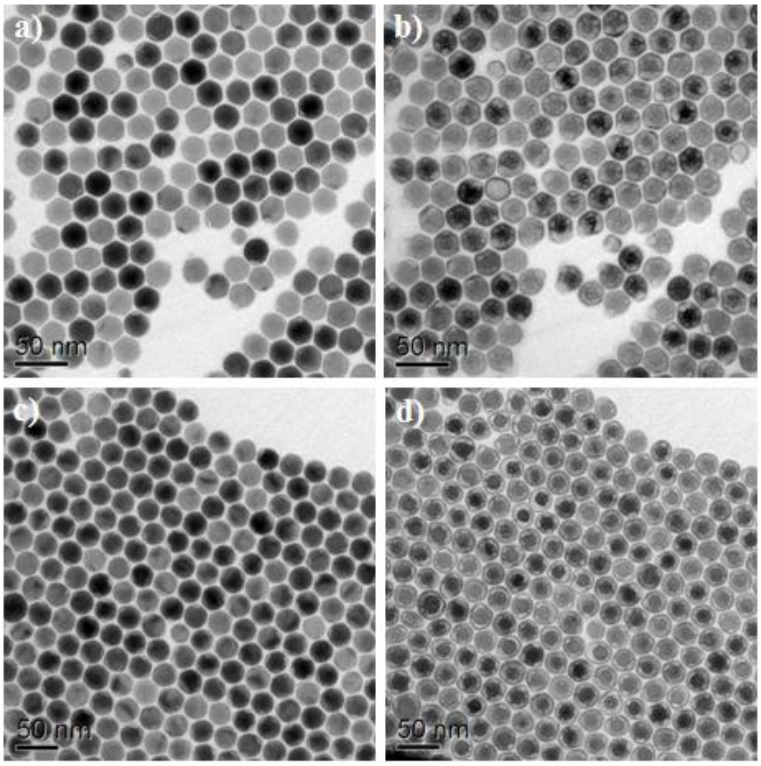

Figure S10. TEM images of core-shell $NaLuF_4$:Gd/Yb/Er@$NaLuF_4$:Nd/Yb NPs a) before and b) after e-beam irradiation for 30 s. c,d) TEM images of core-shell $NaLuF_4$:Gd/Yb/Er@$NaLuF_4$ NPs (27.1 nm) before c) and after d) 30-s e-beam irradiation.

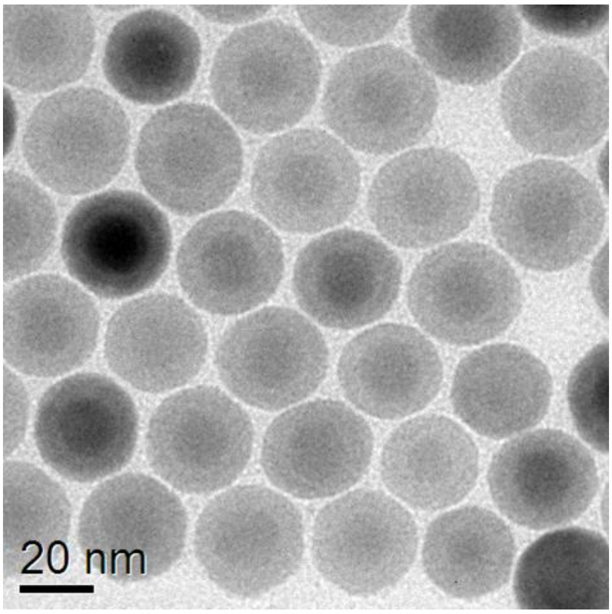

Figure S11. TEM images of $NaLuF_4$:Gd/Yb/Er@$NaYF_4$:Nd/Yb@$NaLuF_4$ NPs after e-beam irradiation for 30 s.

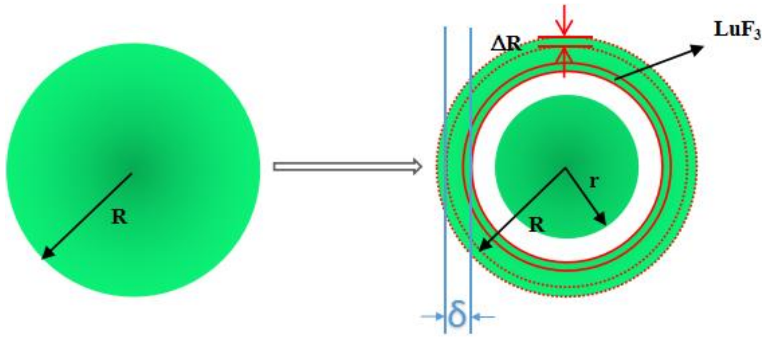

Figure S12. Theoretical calculation of the average size increment ratio for core-shell-shell $NaLuF_4$:Gd/Yb/Er@$NaLuF_4$:Nd/Yb@$NaLuF_4$ NPs after the solid-to-yolk-shell structure conversion. Note that: (i) yolk-shell structure exhibits central symmetry; (ii) during the yolk-shell conversion, $NaLnF_4$ units in crystal lattice are partially decomposed into lanthanide fluoride clusters LnF_x. However, to simplify the calculation, the decomposition process is approximately denoted as $NaLuF_4 \rightarrow LuF_3$, with the neglect of the slight differences between doped ion (Gd, Yb, Er or Nd) and Lu in atomic mass, radius and stacking density. Meanwhile, we presume that the residual LuF_3 units form a thin shell inside the yolk-shell NP. As such, the average size increment ratio (ΔR/R) for core-shell-shell NP after solid-to-yolk-shell conversion was calculated to be 5.9% by the following equation.

$$\Delta R/R = \left[\left[(R\text{-}\delta)^3 - r^3\right] / R^3 \times \frac{\rho(NaLuF_4)}{\rho(LuF_3)} \times \frac{M(LuF_3)}{M(NaLuF_4)} + 1\right]^{1/3} - 1$$

where R (34.1/2 nm), δ (4 nm) and r (18.5/2 nm) are average radius of original solid core-shell-shell NP, thickness of outer shell and radius of retained inner core of yolk-shell NP, respectively; $\rho(NaLuF_4)$ (6.52 g/cm^3) and $\rho(LuF_3)$ (8.44 g/cm^3) are the mass densities of $NaLuF_4$ and LuF_3, respectively; $M(NaLuF_4)$ (273.95) and $M(LuF_3)$ (231.96) are the molecular masses of $NaLuF_4$ and LuF_3 units, respectively. Indeed, the size increment was largely counteracted by the volume reductions caused by the extraction of organics accommodated in lattice as defects and the entering of LnF_x clusters into the interstices of $NaLnF_4$ lattice as interstitial atoms. Under such circumstances, the actual average size increment ratio was far smaller than the theoretically calculated value of 5.9%, namely, the core-shell-shell NP maintained the original outer size after the solid-to-yolk-shell conversion.

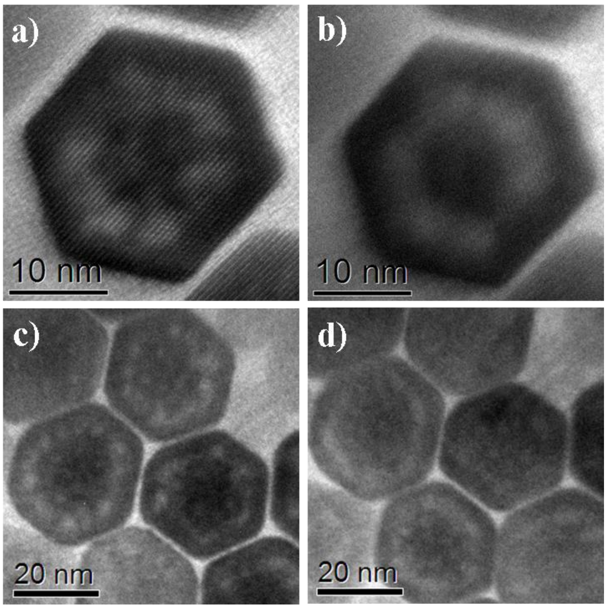

Figure S13. TEM images of core-shell $NaYF_4$:Gd/Yb/Er@$NaYF_4$:Nd/Yb NPs after a) 30-s and b) 60-s e-beam irradiation. TEM images of core/shell $NaGdF_4$:Yb/Er@$NaGdF_4$:Nd/Yb NPs after c) 30-s and d) 60-s e-beam irradiation.

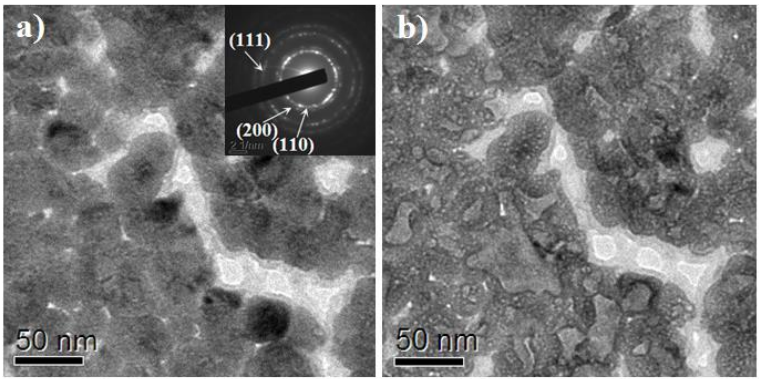

Figure S14. a) TEM image and SAED pattern (inset) of core-shell-shell $NaLuF_4$:Gd/Yb/Er@$NaLuF_4$:Nd/Yb@$NaLuF_4$ NPs after irradiation with fs laser pulses. b) TEM image of NPs shown in a) after e-beam irradiation for 120 s.
